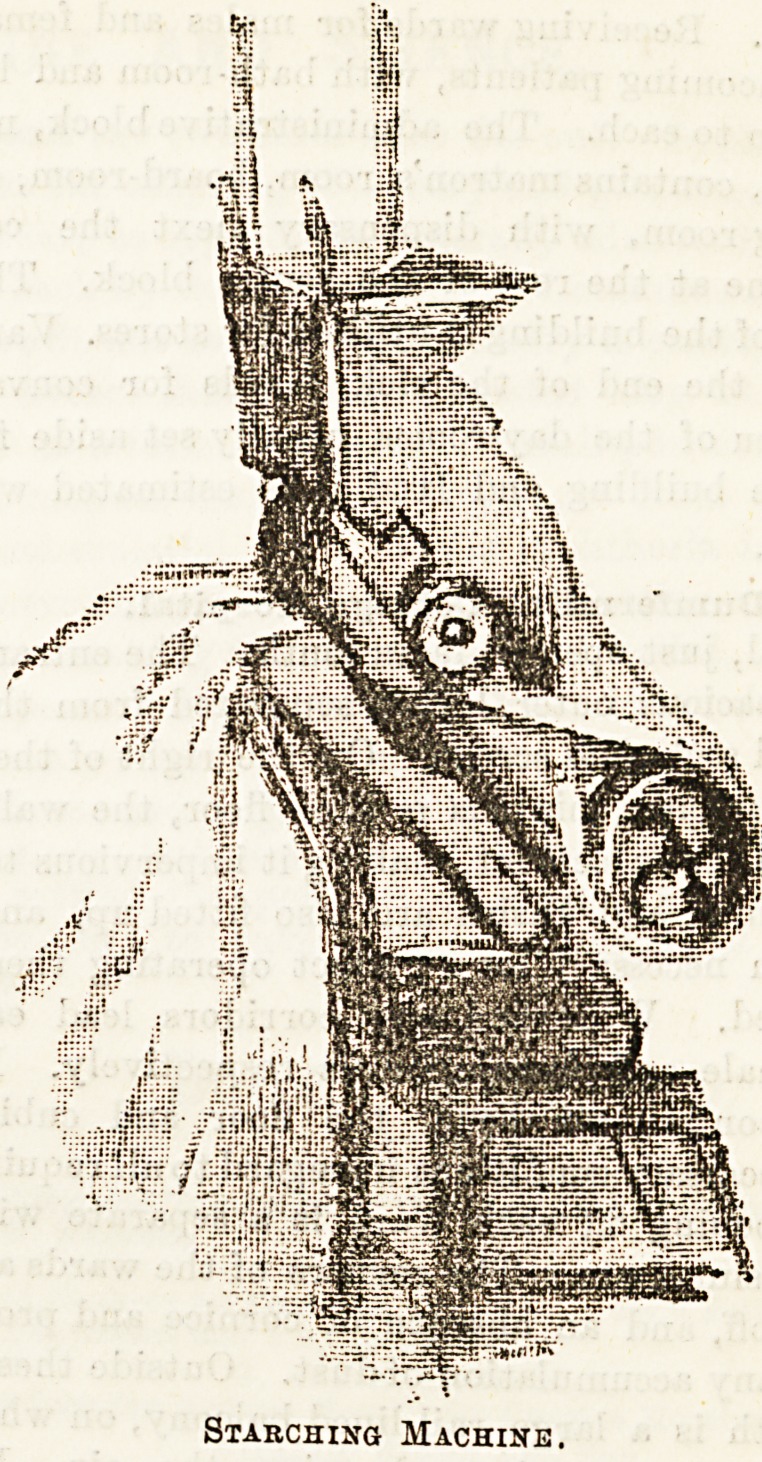# Hospital Laundries

**Published:** 1894-09-29

**Authors:** 


### PRACTICAL DEPARTMENTS.

### HOSPITAL LAUNDRIES (*Continued*).

Such machines as the washers we have described are built for heavy work, and consequently have a very large capacity, those in use at one of the best known American laundries washing at one time as many as 350 ordinary cotton sheets. The time required for a thorough cleansing may vary according to the kind of material to be washed, probably being somewhat longer in a hospital laundry, where a very severe and complete purification is naturally particularly essential.

Taken from the washers in due course the clothes are consigned to an "extractor," a machine which is now familiar enough in modern laundries. The illustration, given by permission of Mr. Armstrong, shows the type of wringer or extractor in use in the Troy Laundries.

The water is driven out of the goods by centrifugal force, the speed reaching 1,600 revolutions per minute. There are various kinds of extractors, a more powerful one yet having been brought out for use in heavy work. The inside cylinder of these extractors or wringers is made of very heavy hammered copper, tinned. It is scarcely necessary, in these days, to point out the great advantage of securing so complete and thorough a "wringing" of the articles in course of washing as this process implies. It is one which has been in use for a number of years in the best steam laundries, but newer and more effective machines are constantly coming to the front. Instead of the tedious business of hand-wringing, necessitating a very large staff of workers, some fifty sheets are thus in the shortest possible space of time reduced to a state of comparative dryness, which seems little short of marvellous. This thorough wringing is especially good, because clothes ironed while very wet are far harder and harsher than when the water has been forced well out of them in this manner.

The third process is to remove the contents of the extractors to the "tumblers," machines somewhat similar in construction to the "washers," and designed to loosen the clothes, which are turned out from the wringing process necessarily matted together. The tumblers have the same automatic reversing movement. They are made of perforated brass, and by introducing a strong blast of hot air into the

WRINGING MACHINE.

cylinder (the perforation giving free escape for the vapour) the remaining moisture is quickly taken out of the linen, and the shaking-out thus performed at the same time saves much in the direction of labour.

STARCHING MACHINE.

Mangling and ironing are the final processes, but a consideration of these must wait till our next article. The second illustration given here shows a "starching machine," which facilitates to a considerable extent this important branch of laundry work. The sketch is given by kind permission of the managers of the very excellent Troy Laundry at Ealing.

## Figures and Tables

**Figure f1:**
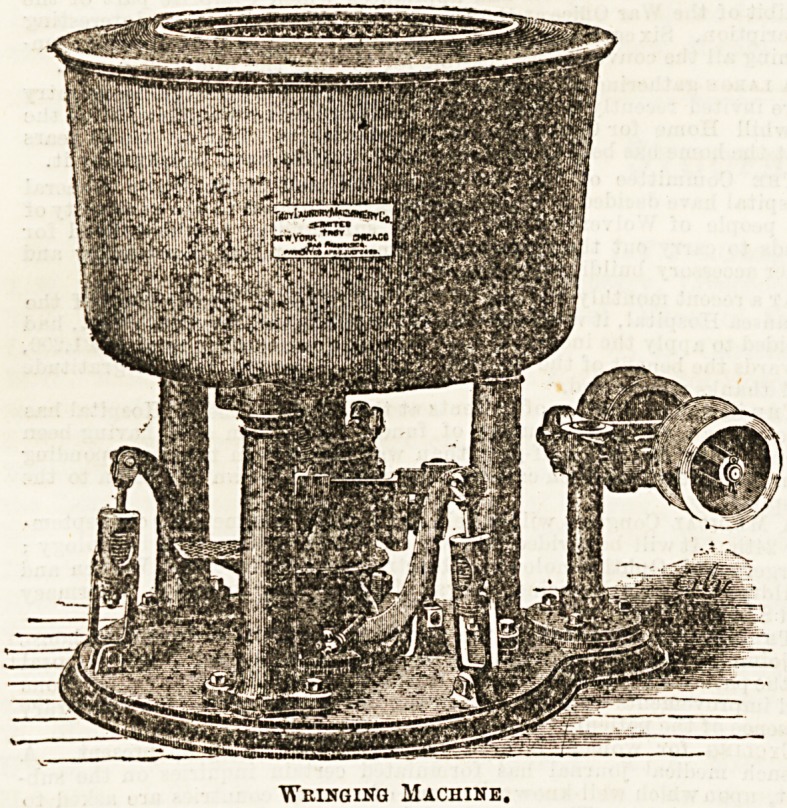


**Figure f2:**